# ACOT12-mediated acetyl-CoA hydrolysis suppresses intrahepatic cholangiocarcinoma metastasis by inhibiting epithelial-mesenchymal transition

**DOI:** 10.7150/jca.62169

**Published:** 2022-03-14

**Authors:** Xu Zhou, Yu Zhou, Weiqing Shao, Liang Hong, Ming Lu, Wenwei Zhu

**Affiliations:** 1 Department of General Surgery, Huashan Hospital, Fudan University, 12 Urumqi Road (M), Shanghai 200040, China.; 2 Department of Infection, Rui'an People's Hospital, 108 WanSong Road, Rui'an, Wenzhou, Zhejiang 325200, China.; 3 Shanghai Institute of Nutrition and Health, Chinese Academy of Sciences, Shanghai, 200031, China

**Keywords:** ICC, ACOT12, cancer metastasis, Acetyl-CoA, histone acetylation, EMT

## Abstract

Intrahepatic cholangiocarcinoma (ICC) is the most common malignant bile duct tumor in the liver and the second most common primary liver cancer with increasing morbidity and poor prognosis. Metabolic aberration plays key roles in cancer progression. As a key metabolic intermediate, acetyl-CoA accumulation shows close association with cancer metastasis. However, the role of acetyl-CoA metabolic aberration in ICC is still undetermined. Here, by investigating tissue samples from ICC patients and ICC cell lines, we found that acyl-CoA thioesterase 12 (ACOT12) expression is significantly down-regulated in ICC tissues, and is associated with poor prognosis of ICC. *In vitro* and *in vivo* studies demonstrated that ACOT12 suppressed ICC cells metastasis. Further mechanistic studies revealed that down-regulation of ACOT12 promoted ICC metastasis by inducing Slug expression and epithelial-mesenchymal transition (EMT). Our findings link ACOT12-regulated-acetyl-coA metabolic aberration with ICC metastasis and imply that ACOT12 could be a prognostic marker and a potential therapeutic target for ICC metastasis.

## Introduction

Intrahepatic cholangiocarcinoma (ICC) is the most common malignant bile duct tumor in the liver and the second most common primary liver cancer, accounting for 10-20% of primary liver cancer^1^, ^2^. In the past decades, the morbidity and mortality of ICC have been both increasing rapidly worldwide^3^. However, most ICC patients were diagnosed at advanced stages, only a small part of ICC patients are eligible for surgical intervention^4^, ^5^. Furthermore, the recurrence rate of ICC patients after resection remains very high, usually between 67% and 75%^6^, and the 5-year survival rate is lower than 10%^7^. Molecular biology of ICC pathogenesis may provide effective treatment strategies and improve patient outcomes^8^. Therefore, it is urgent to understand the underlying mechanism and search optional therapeutic targets.

Tumor cells are known to undergo metabolic reprogramming to cope with the increased metabolic demands for an uncontrolled malignant phenotype. These metabolic alterations participate in signaling in various aspects of tumor cell biology. One of the most important findings is the link between metabolism and the regulation of the epigenome, where acetyl-CoA has a main role as an essential cofactor in the post-translational acetylation reactions of histones^9^, ^10^. In mammalians, acetyl-CoA not only the initiated metabolite of tricarboxylic acid (TCA) cycle, but also the precursor of lipid biosynthesis. On the other hand, as the substrate for acetylation reactions, acetyl-CoA plays an important role in epigenetic regulation as its dynamic association with the acetylation of histones^11^. Acetyl-CoA can be hydrolyzed by acyl-CoA thioesterase 12 (ACOT12)^12^, and the other enzymes responsible for regulation of acetyl-CoA, including adenosine triphosphate (ATP)-citrate lyase (ACLY), acetyl-CoA synthetase 1 (ACSS1), acetyl-CoA synthetase 2 (ACSS2), pyruvate dehydrogenase complex (PDC) and acyl-CoA carboxylase1 (ACC1), are reported to participate in tumor growth and progression^13^-^17^.

Tumor metastasis consists of sequential, interlinked, and selective steps^18^, many steps are favored by epithelial-to-mesenchymal transition (EMT), which is a key driver of cancer metastasis^19^. The EMT is a developmental program exploited by cancer cells to transition from a sessile epithelial state to a motile, invasive mesenchymal state, facilitating escape from the primary tumor^20^. The EMT process has been reported in multiple cancers including breast cancer^21^, prostate cancer^22^ and colorectal cancer^23^. Acetyl-CoA metabolic aberrations have been reported to play an important role in cancer metastasis via regulating EMT^24^-^26^. However, the role of acetyl-CoA metabolic aberration in ICC is still undetermined.

In this study, we examined the ACOT12 expression in ICC tissues and its associations with clinical prognosis of ICC, and revealed the role and the mechanism of down-regulated expression of ACOT12 in ICC metastasis.

## Materials and Methods

### Tissue Samples

ICC patients tissue samples were collected from Huashan Hospital Affiliated to Fudan University (Shanghai, China). Inclusion criteria were: Patients with primary diagnosis of ICC between 2014 and 2017, which underwent surgical resection and pathologically proven ICC. Exclusion criteria were: Patients who received neoadjuvant treatment before primary surgery were excluded. The tissues were first treated with liquid nitrogen. Then the tissues were stored at -80 °C. Informed consents were obtained from patients, and the experimental protocols were agreed with the Ethics Committee of Fudan University. All patients were diagnosed independently by three pathologists. Data from mRNA expression microarray analysis for 36 ICC and 9 normal tissues were downloaded from The Cancer Genome Atlas (TCGA) database (http://tcga-data.nci.nih.gov/).

### Cell lines and culture conditions

The human ICC cell lines RBE, CCLP1, HCCC9810, HUCCT1 were purchased from the Chinese Academy of Sciences Cell Bank (Shanghai, China). Cells were maintained in DMEM medium (Gibco, USA) with 10 % fetal bovine serum (Biological Industries, Israel), penicillin/ streptomycin 100 units/mL at 37°C in a humidified incubator containing 5% CO2.

### Drug Treatments

For TSA treatment, cells were treated with 0.5 mM for 24 h. For C646 treatment, cells were treated with 10 mM for 24 h. Both TSA and C646 were obtained from Selleck.

### Plasmid construction and transfection

ACOT12 lentiviral overexpression vector was obtained by cloning human ACOT12 coding sequence into pCDH vector. Slug lentiviral overexpression vector was obtained by cloning human Slug coding sequence into pCDH vector. The shRNA sequences for target genes in this paper were purchased from Sigma-Aldrich. The sequences of shRNAs targeting for ACOT12 are as follows:

shACOT12-1: 5'- GGATGATTTCAGACGCTATCG -3'; shACOT12-2: 5'- GCAAACCATCACGGAAATACA -3'; shACOT12-3: 5'- GCTGGAGTTTCCTGCGTTACA -3'; shSlug-1: 5'- CCGAAGCCAAATGACAAATAA -3'; shSlug-2: 5'- GCGCCCTGAAGATGCATATTC -3'.

### RNA Preparation and qRT-PCR

RNA isolation and cDNA obtaining were performed as described previously^27^. For qRT-PCR analysis, cDNA was amplified using SYBR Green Realtime PCR Master Mix (Takara, Japan). qRT-PCR reactions were performed in triplicates as the following conditions: 95 °C/20 s, 40 cycles of 95 °C/60 s and 60 °C/20 s on using the ABI PRISM 7900 Sequence Detection System (Applied Biosystems, Foster City, CA, USA) and repeated at least three times. Relative mRNA levels were analyzed by the -ΔΔCt method using β-actin as the endogenous control and presented as 2^^-ΔΔCt^. Primer sequences are as follow:

Actin-F/R: CATGTACGTTGCTATCCAGGC/CTCCTTAATGTCACGCACGAT, ACOT12-F/R: GAGGAAGGAGCGGTTTCCAC/AGGCGGCTTGCAGAAATAGTA, Slug- F/R: CGAACTGGACACACATACAGTG/CTGAGGATCTCTGGTTGTGGT.

### Western blot analysis

Protein extraction and western blotting were performed as described previously^28^. Briefly, cells were lysed on ice for 30 min in radio-immunoprecipitation assay buffer. The lysates were centrifuged at 12,000 rpm at 4°C for 15 min, the supernatants were collected, and protein concentrations were determined using bicinchoninic acid assay TIANGEN, Beijing, China). Equal amounts of protein were separated by 10% SDS-PAGE followed by electrotransfer onto a polyvinylidene difluoride membrane (Thermo Fisher Scientific, MA, USA). Membranes were blocked with 5% nonfat milk for 2 h and then incubated overnight at 4°C with diluted (1:1000) primary antibodies against MMP2, MMP9, ZEB1, Snail, Slug, Twist and β-actin were obtained from Cell Signaling Technology (MA, USA), and ACOT12, N-Cadherin, E-Cadherin, histone H3, histone H3ac, histone H4 and histone H4ac obtained from Abcam followed by incubation with the secondary antibody (1:5000, Cell Signaling Technology). An electrochemiluminescence detection system (GE, USA) was used for signal detection.

### Immunohistochemistry (IHC) staining

Immunohistochemical staining was performed on TMA slides by two steps. After deparaffinage and rehydration, the tissue slides were treated with 0.01mol/L sodium citrate (pH 6.0) in a microwave oven for 10 minutes for antigen retrieval. Then, sections were incubated with the monoclonal anti-ACOT12 or anti-ACSS1or anti-ACLY antibodies (1:100) at 4°C overnight and subsequently developed using ChemMate DAKO EnVision Detection Kit, Peroxidase/DAB, Rabbit/Mouse (Dako Cytomation, Glostrup, Denmark). Later, slides were counterstained with hematoxylin. All procedures were conducted consistently only with omitting primary antibody in negative controls. Immunohistochemistry staining was assessed by two independent pathologists with no prior knowledge of patient characteristics. Discrepancies were resolved by consensus. The staining extent score was on a scale of 1 to 4, corresponding to the percentage of immunoreactive tumor cells and the staining intensity. Score 1 is defined as negativity or scatter positivity. Score 2 is defined as low intensity, diffused positivity. Score 3 is defined as continuous positivity. Score 4 is defined as high positivity.

### Cell Proliferation Assay

Cell proliferation was measured by using Cell Counting Kit-8 (Dojindo, Kumamoto, Japan). ICC cell lines were seeded into 96-well plates at a density of 1×10^3^ cells per well in a final volume of 100 μL medium containing 10% FBS. Then cells were incubated at 37 °C in 5% CO_2_ for 24, 48, 72, and 96 hours and the medium was replaced with 10 μL CCK-8 solution and 90 μL of fresh medium each well. The absorbance at 450 nm was measured after incubation for 1 hours at 37°C in 5% CO_2_. Each experiment was repeated for three times on each condition.

### Colony formation assay

ICC stable cell lines were seeded in six-well plates at a density of 8 × 10^2^ cells/well. Cells were incubated at 37 °C in 5% CO_2_ for about 10-14 days, then cells were fixed in 4% formalin for 20 minutes and then stained with 0.5% crystal violet after washed twice with PBS. After that cells were allowed to air dry at room temperature. The number of colonies was counted.

### Wound-Healing Assay

ICC cell lines were cultured in monolayers in a six-well plate format overnight and wounded with a pipette tip. Then images were taken at 0 hour and 24 hours with light microscope.

### Invasion Assay

To assay the ICC cell lines' ability of invasion, 24-well transwell chambers, with upper and lower culture compartments separated by polycarbonate membranes with 8 μm pores (BD Pharmingen). The bottom chamber was filled with 10% FBS supplemented DMEM. 4×10^4^ cells with prepared for invasion 1 hour after seeding 100 μL matrigel in the upper chamber. Then cells suspended in serum-free medium were then seeded into the upper chamber and maintained in normal cell culture condition mentioned above. After 48 hours, cells that invaded to the underside of the membrane were stained with crystal violet, calculated with light microscope.

### Acetyl-coA measurement

Cells seeded in 10 cm dishes were grew to 80% of confluence, then washed with cold PBS, harvested in RIPA buffer, and sonicated. The cellular protein was precipitated with PCA (perchloric acid). After centrifugation, supernatant was recovered and neutralized by the addition of potassium bicarbonate until the pH of the sample was in the range of 6-8 and precipitates were removed by centrifugation. The supernatant was used to determine acetyl-CoA concentration in triplicate by using an acetyl-CoA assay kit (Sigma MAK039) according to manufacturer's instructions.

### *In vivo* Tumor Metastasis Assays

Five-week-old male nude mice (BALB/c/nu/nu) were housed and randomly divided into indicated groups (6 mice/group) before inoculation, and double-blinded evaluation was performed when measuring tumor weight, volume, and number of metastatic nodules. Different stable ICC cell lines (HUCCT1-OENC, ACOT12-OE, ACOT12-OE and Slug-OE,) were injected into the intraperitoneal cavity of nude mice (2.5x10^6^cells/mouse in 200 μL PBS) to establish metastatic model and (RBE-shNC, shACOT12, shACOT12 and shSlug) injected into the liver (2.5x10^6^cells/mouse in 50 μL l PBS) to establish orthotopic model. All mice were sacrificed 6 weeks later and their tumor, lung and abdominal cavity tissues were removed and analyzed. Above tissues were fixed with paraformaldehyde (4%) before dehydration and embedding in paraffin. Lung tissues were stained with H&E according to standard protocols and the number of lung metastasis was calculated and evaluated under microscope.

### Statistical analysis

All experiments were performed in triplicate and means and standard error of the mean or standard deviation were subjected to the Student's t-test for pairwise comparison or ANOVA for multivariate analysis. Kaplan-Meier survival analysis was performed using Graphpad Prism 5 software. Significance level was set at P < 0.05 for all tests.

## Results

### ACOT12 is down-regulated in ICCs

**Figure 1:**
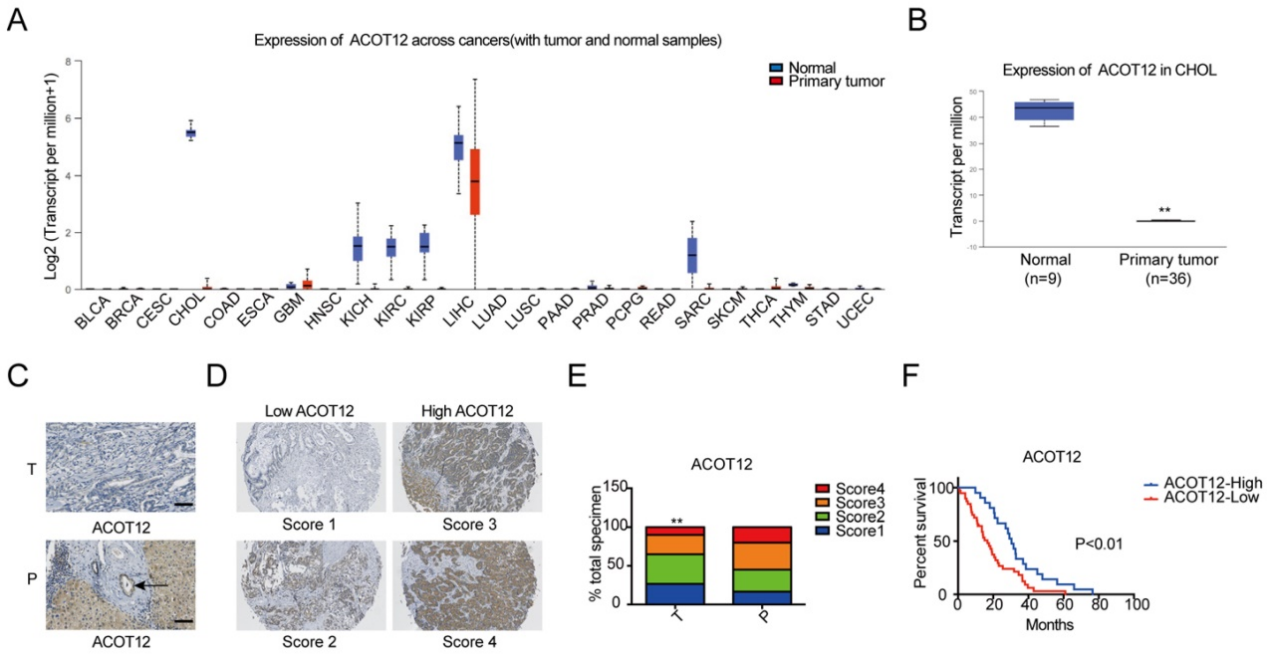
** ACOT12 is downregulated and a key gene in ICC.** (A) Expression of ACOT12 across cancers with tumor and normal tissues from TCGA database. (B) Expression of ACOT12 in 36 ICC tissues and 9 normal tissues from TCGA database. Significance was determined using the Student's t test. (C) Representative images of immunohistochemical (IHC) staining of ACOT12 from one of 60 paired samples of ICC tissues (T) and para-tumor tissues (P) from a tissue microarray. Scale bar, 50μm. (D) Scores indicate ACOT12 protein levels in representative tumor tissues. (E) Quantification of ACOT12 protein levels according to IHC scores in 60 paired samples of T and P, respectively. Significance was determined using the χ2 test. Data are shown as percentage of total specimen. (F) The prognostic significance of ACOT12 for ICC patients from a tissue microarray analyzed by Kaplan-Meier survival curves. A log-rank test was used to assess the statistical significance of differences. (*P< 0.05, **P< 0.01).

To investigate the role of acetyl-CoA metabolic alteration in ICC, we firstly evaluated the expression level of acetyl-CoA hydrolysis gene ACOT12 across cancers with tumor and normal samples from the TCGA public database, and found that ACOT12 down-regulated most significantly in ICC (Fig. 1A, B). We further validated the association of ACOT12 expression with ICC, as well as their prognostic values in human ICCs, by performing tissue microarray-based IHC studies using ICC tissues from 60 patients. We found that the expression level of ACOT12 was lower in ICC tissues than para-tumor tissues (Fig. 1C, E). According to the immunostaining scores for ACOT12, these patients were divided into high (score 3-4) or low (score 1-2) expression group (Fig. 1D). Low expression of ACOT12 was found to be significantly associated with reduced overall survival (OS) (Fig. 1F). We also evaluated other genes responsible for regulation of acetyl-CoA in paired tissues of ICC and normal tissues from TCGA database. The results showed that the expression levels of ACLY, ACSS1, ACC1 were higher in ICC tissues compared with those in normal tissues, and the expression level of ACSS2 was lower in ICC tissues than that in normal tissues while PDC had no statistical difference (Fig. S1A-E). We chose ACLY and ACSS1, which changed most significantly in ICC of five other genes responsible for regulation of acetyl-CoA, to validate the association of their expression with ICC, as well as their prognostic values in human ICCs, by performing tissue microarray-based IHC studies using ICC tissues from 60 patients. We found that the expression level of ACLY was higher in ICC tissues than para-tumor tissues (Fig. S1F, H), while the expression level of ACSS1 had no differences between ICC tissues and para-tumor tissues (Fig. S1J, L). According to the immunostaining scores for ACLY and ACSS1, these patients were divided into high (score 3-4) or low (score 1-2) expression group (Fig. S1G, K), and both of them had no significant association with OS (Fig. S1I, M). These results indicate that ACOT12 is down-regulated in ICC and is correlated with poor prognosis of ICCs.

### ACOT12 suppresses the migration and invasion of ICC cells *in vitro*

**Figure 2:**
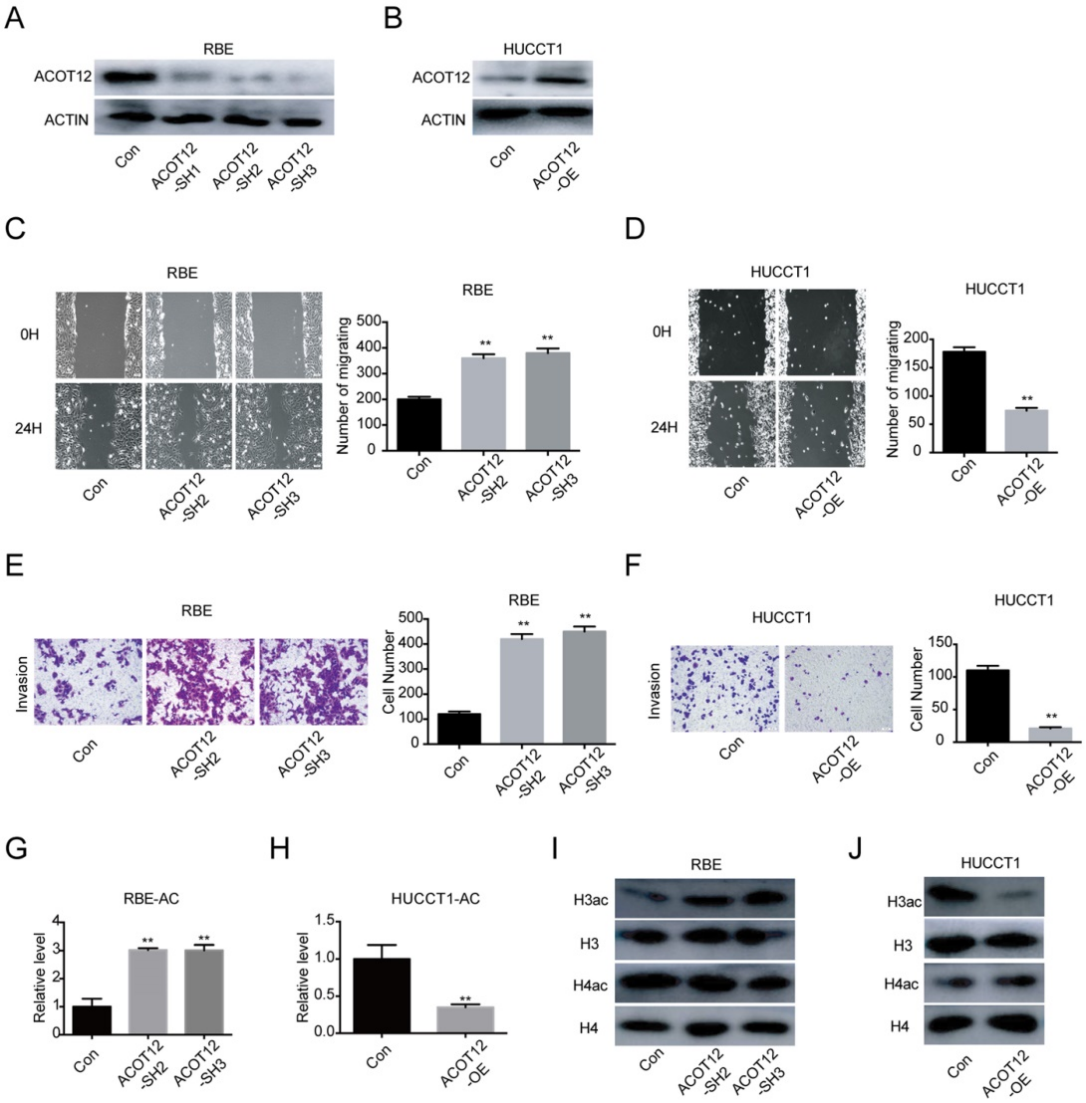
** ACOT12 inhibits migration and invasion of ICC cells.** (A, B) Knockdown or overexpression of ACOT12 in ICC cells was detected by WB. (C, D) The effects of ACOT12 gain- or loss-of-function on migration of ICC cells were assessed by wound healing assay. (E, F) The effects of ACOT12 gain- or loss-of-function on invasion of ICC cells were assessed by transwell assay. (G, H) The effects of ACOT12 gain- or loss-of-function on the acetyl-coA levels of ICC cells. (I, J) The effects of ACOT12 gain- or loss-of-function on histone H3 acetylation (H3ac) and histone H4 acetylation (H4ac) levels of ICC cells were detected by WB. SH: shRNA, OE: Overexpression, Data are shown as mean ± SD, significance was determined using the Student's t test. (*P< 0.05, **P< 0.01).

To investigate the role of ACOT12 in ICC progression *in vitro*, we firstly detected the ACOT12 expression by qRT-PCR and WB in 4 ICC cells (Fig. S2A, B). According to the results that RBE and CCLP1 had high ACOT12 expression, HCCC9810 and HUCCT1 had low ACOT12 expression, we established ACOT12 knockdown cell in RBE cells (Fig. S2C, 2A) and ACOT12 overexpression cell in HUCCT1 cells (Fig. S2D, 2B). ACOT12 knockdown or overexpression did not change cells proliferation as determined by the CCK-8 and colony formation assays (Fig. S3). While, the wound healing assay revealed that cell migration in ICC was significantly promoted by ACOT12 down-regulation and impeded by ACOT12 overexpression (Fig. 2C, D). Similar results of cell invasion could also be obtained by using the trans-well assay (Fig. 2E, F). These *in vitro* results indicate that ACOT12 plays important roles in suppressing migration and invasion of ICC cells.

### A decrease in histone acetylation is required for the anti-metastatic effect of ACOT12

As ACOT12 is responsible for hydrolyzing acetyl-CoA into acetate and coenzyme A (CoA), we then examined the effects of modulating ACOT12 expression on cellular acetyl-CoA levels, and found that the cellular acetyl-CoA level was increased in ACOT12 knockdown cells, and was reduced in ACOT12 overexpression cells (Fig. 2G, H). Next, as histone acetylation is dynamically affected by the cellular acetyl-CoA pool, we then determined the histone acetylation levels, and found that ACOT12 silencing led to a significant increase of H3ac level (H3ac) level (Figure 2I), while ACOT12 overexpression resulted in a dramatic decrease of histone H3 acetylation (Figure 2J). These results indicate that ACOT12 regulates the acetyl-CoA levels and H3 acetylation in ICC cells.

**Figure 3:**
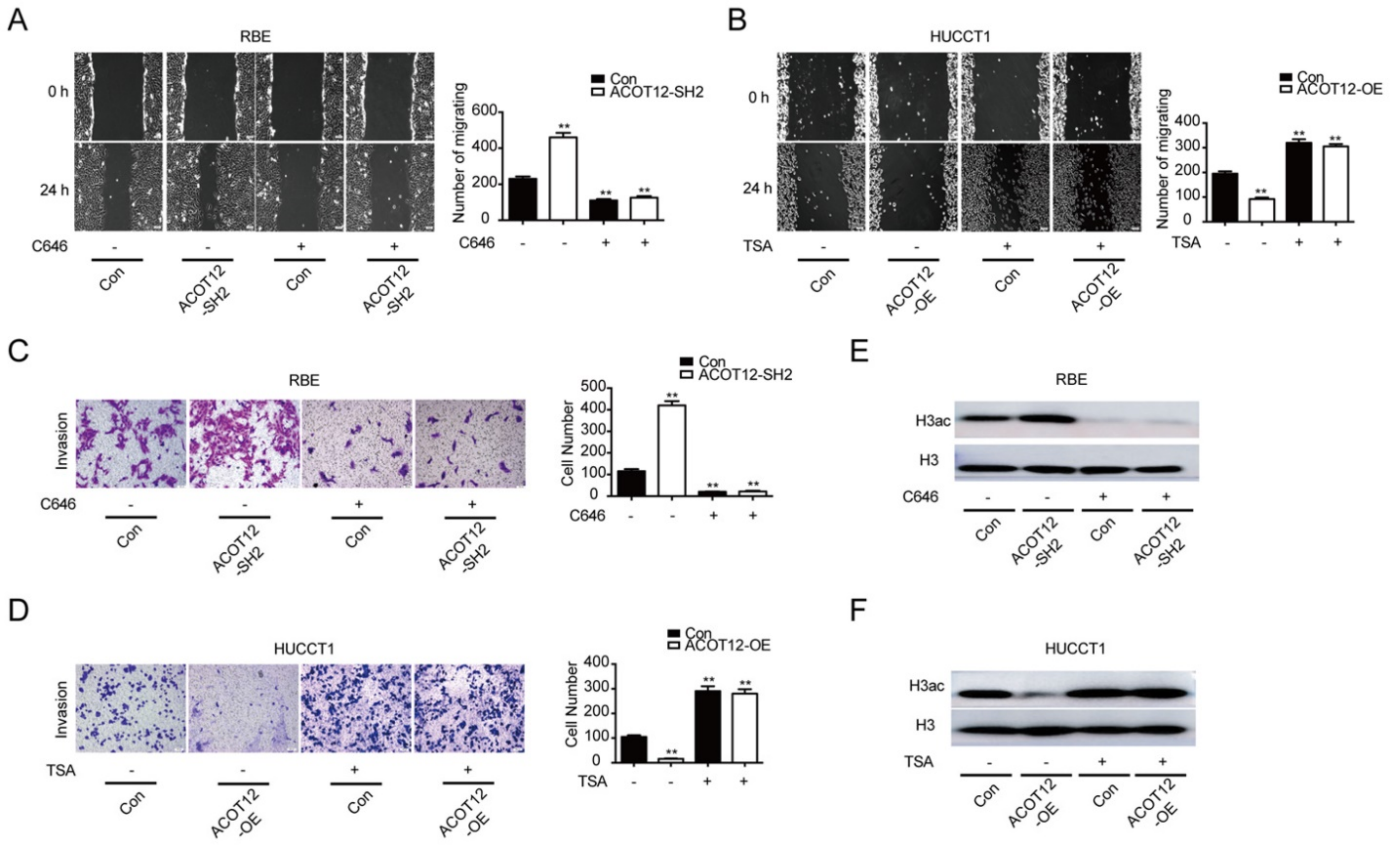
** A decrease in histone acetylation is required for the anti-metastatic effect of ACOT12**. (A, B) The effects of C646 or TSA on migration of ACOT12 knockdown or overexpression ICC cells were assessed by wound healing assay. (C, D) The effects of C646 or TSA on invasion of ACOT12 knockdown or overexpression ICC cells were assessed by transwell assay. (E, F) The effects of C646 or TSA on H3ac level of ACOT12 knockdown or overexpression ICC cells were detected by WB. SH: shRNA, OE: Overexpression, Data are shown as mean ± SD, significance was determined using the Student's t test or one-way ANOVA. (*P< 0.05, **P< 0.01), comparisons between the first group and other three groups.

To verify whether ACOT12-dependent change of histone acetylation is required for the anti-metastatic effect of ACOT12, we treated cells with C646, a p300 acetyltransferase inhibitor, or Trichostatin A (TSA), a histone deacetylase (HDAC) inhibitor, to modulate histone acetylation levels. We found that C646 treatment hindered the effect of ACOT12 knockdown on promoting the cell migration and invasion ability (Fig. 3A, C), and H3ac expression (Fig. 3E). On the other hand, TSA treatment hindered the effect of ACOT12 overexpression on suppressing the cell migration and invasion ability (Fig. 3B, D), and H3ac expression (Fig. 3F). Collectively, these data demonstrate that decreases in acetyl-CoA level and H3 acetylation are required for the anti-metastatic effect of ACOT12.

### ACOT12 suppresses epithelial-mesenchymal transition of ICC cells by inhibiting Slug expression

**Figure 4:**
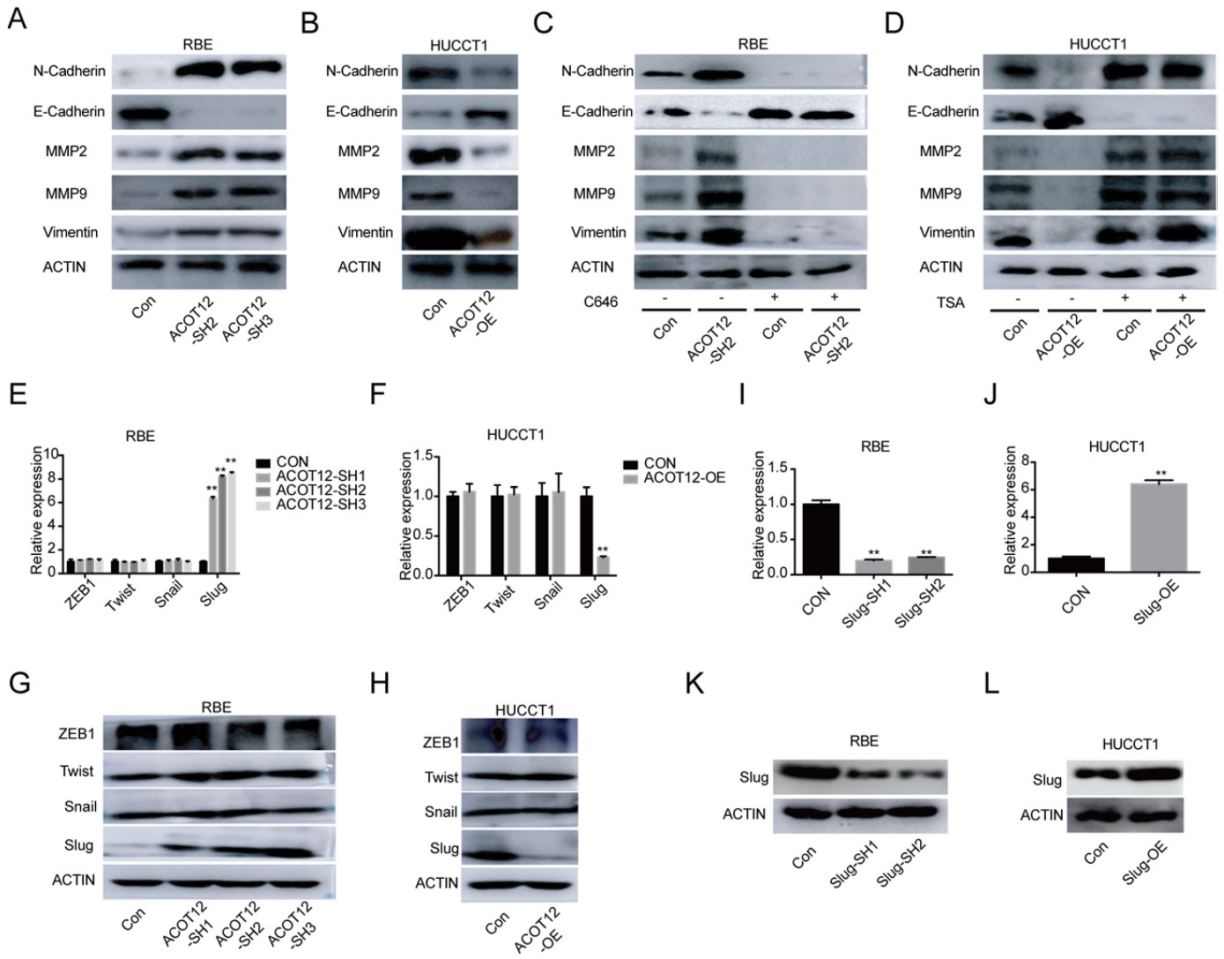
** ACOT12 suppresses Slug expression in ICC cells**. (A, B) The effects of ACOT12 gain- or loss-of-function on expression of EMT-related genes of ICC cells were detected by WB. (C, D) The effects of C646 or TSA on expression of EMT-related genes of ACOT12 knockdown or overexpression ICC cells were detected by WB. (E, F) The effects of ACOT12 gain- or loss-of-function on expression of Slug of ICC cells were detected by qRT-PCR. (G, H) The effects of ACOT12 gain- or loss-of-function on expression of Slug of ICC cells were detected by WB. (I, J) Knockdown or overexpression of Slug in ICC cells was detected by qRT-PCR. (K, L) Knockdown or overexpression of Slug in ICC cells was detected by WB. SH: shRNA, OE: Overexpression, Data are shown as mean ± SD, significance was determined using the Student's t test. (*P< 0.05, **P< 0.01).

To investigate the mechanism underlying the suppressive effects of ACOT12 on ICC metastasis, we detected the expression of the key genes in epithelial-mesenchymal transition (EMT), including N-cadherin, E-cadherin, MMP2, MMP9 and Vimentin by WB. The results showed that the protein levels of N-cadherin, MMP2, MMP9 and Vimentin were increased, and the protein levels of E-cadherin expression was reduced when ACOT12 is knocked down in RBE cells (Fig. 4A). While after ACOT12 was overexpressed, the protein levels of N-cadherin, MMP2, MMP9 and Vimentin expression were reduced and E-cadherin expression was elevated in HUCCT1 cells (Fig. 4B). Furthermore, we found that C646 treatment hindered the effect of ACOT12 knockdown on increasing E-cadherin expression, and on reducing the expression of N-cadherin, MMP2, MMP9, Vimentin (Fig. 4C). On the other hand, TSA treatment hindered the effect of ACOT12 overexpression on reducing E-cadherin expression, and on increasing N-cadherin, MMP2, MMP9, Vimentin (Fig. 4D).

**Figure 5:**
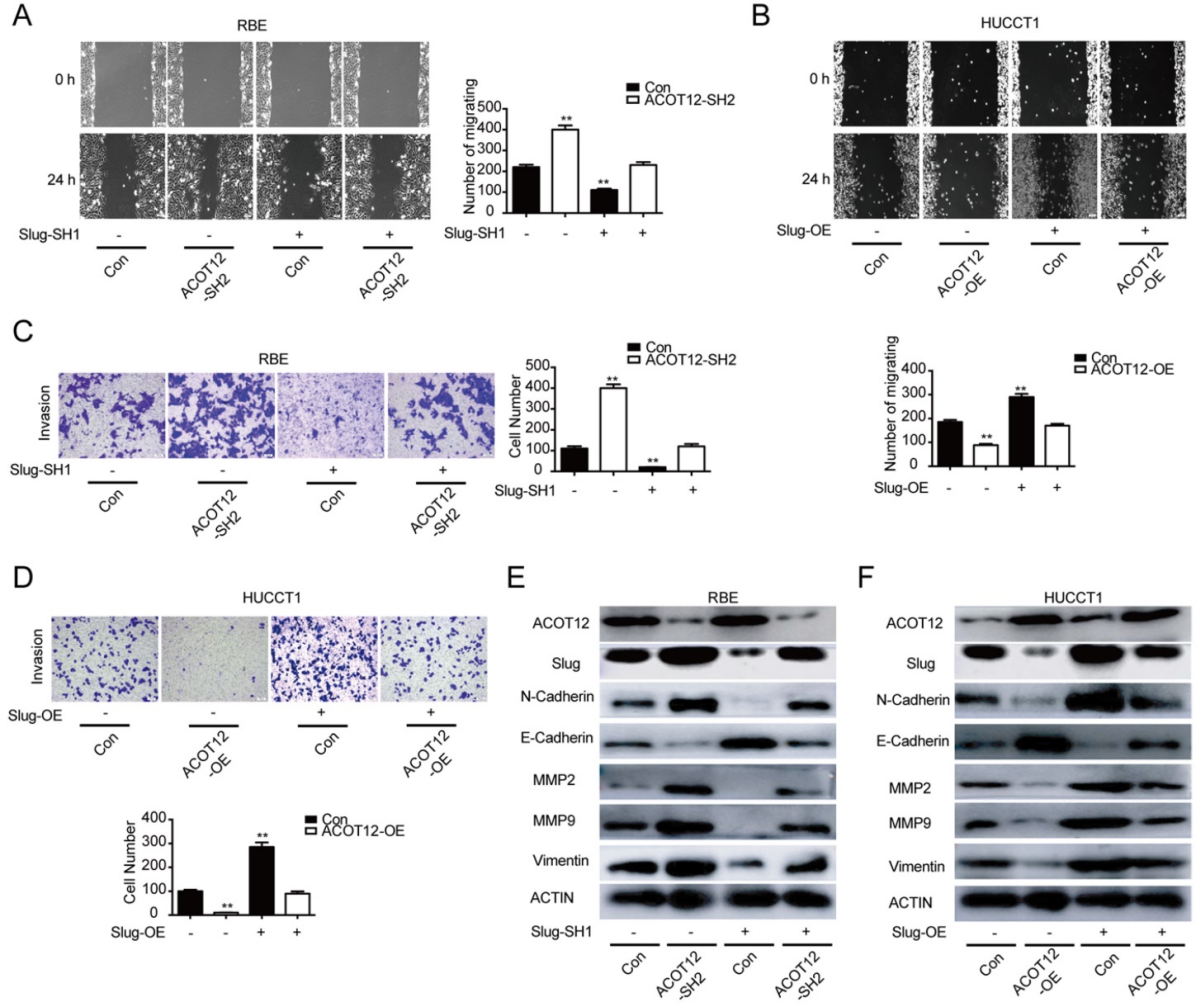
** ACOT12 suppresses ICC cells metastasis via inhibiting Slug *in vitro*.** (A, B) The effects of Slug gain- or loss-of-function on migration of ACOT12 knockdown or overexpression ICC cells were assessed by wound healing assay. (C, D) The effects of Slug gain- or loss-of-function on invasion of ACOT12 knockdown or overexpression ICC cells were assessed by transwell assay. (E, F) The effects of Slug gain- or loss-of-function on expression of EMT-related genes of ACOT12 knockdown or overexpression ICC cells were detected by WB. SH: shRNA, OE: Overexpression, Data are shown as mean ± SD, significance was determined using the Student's t test or one-way ANOVA. (*P< 0.05, **P< 0.01), comparisons between the first group and other three groups.

To find which EMT-related genes play an important role in ACOT12 inhibitory effect on the metastasis of ICC cells. qRT-PCR and WB were used to detect the effect of ACOT12 on the expression of EMT-related genes, ZEB1, Twist, Snail and Slug. Results indicated that only Slug was up-regulated in ACOT12 konckdown cell (Fig. 4E, G), while down-regulated in ACOT12 overexpression cell (Fig. 4F, H). To explore whether ACOT12 suppresses ICC metastasis by down-regulating Slug expression, we stably knocked down Slug in ACOT12-knockdown RBE cells or reexpressed Slug in ACOT12-overexpression HUCCT1 cells (Fig. 4I-L). We found that inhibition of Slug hindered the effect of ACOT12 knockdown on promoting the cell migration and invasion ability (Fig. 5A, C), and promoted E-cadherin expression, inhibited N-cadherin, MMP2, MMP9 and Vimentin (Fig. 5E). On the other hand, reexpression of Slug hindered the effect of ACOT12 overexpression on suppressing the cell migration and invasion ability (Fig. 5B, D), and inhibited E-cadherin expression, promoted N-cadherin, MMP2, MMP9 and Vimentin (Fig. 5F). Together, these results indicate that ACOT12 suppresses epithelial-mesenchymal transition of ICC cells by inhibiting Slug expression.

### ACOT12 suppresses ICC metastasis *in vivo*

**Figure 6:**
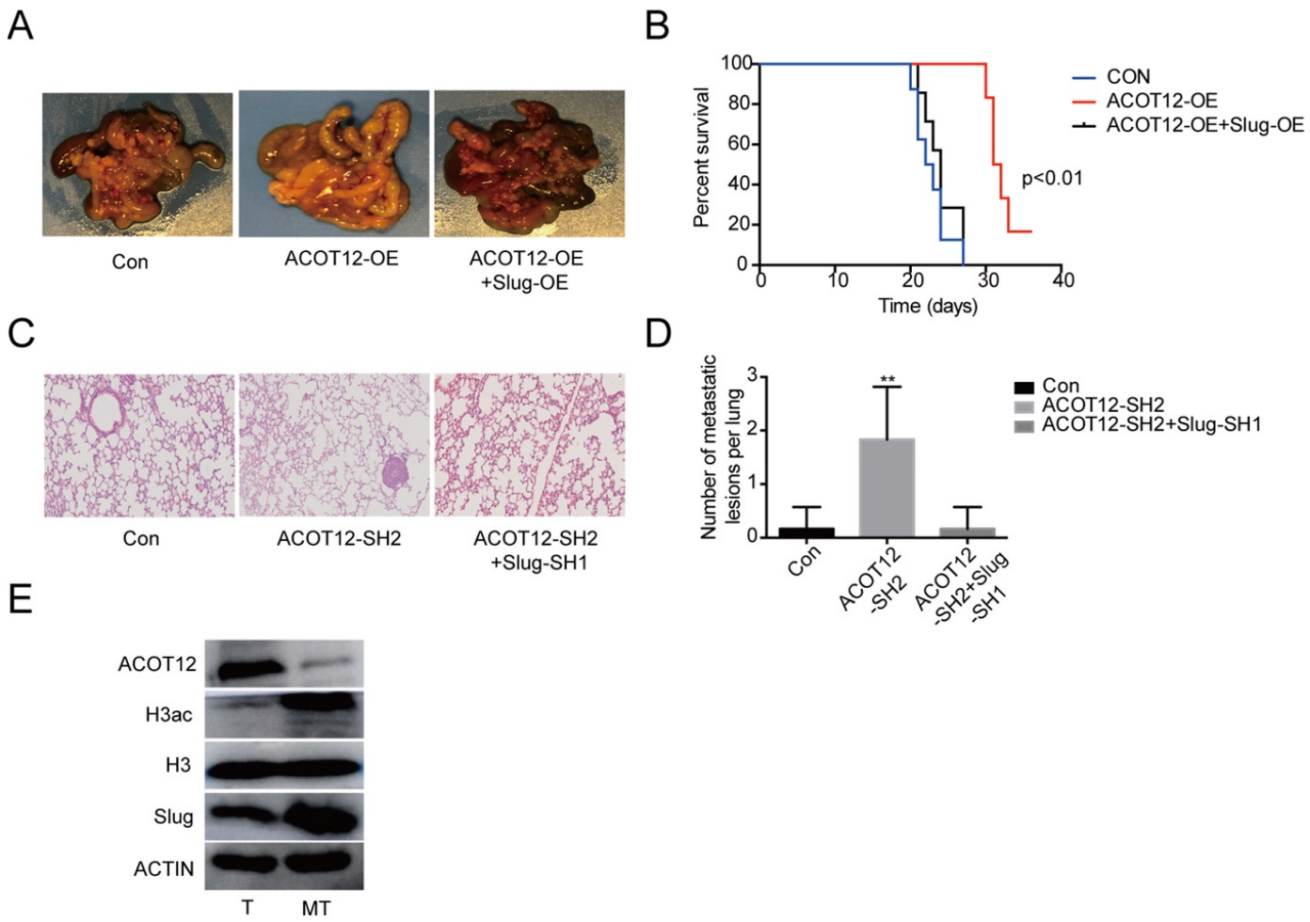
** ACOT12 suppresses ICC cells metastasis via inhibiting Slug *in vivo***. (A)The effect of ACOT12 overexpression on cell metastasis and the effect of Slug on ACOT12 overexpression cell metastasis were assessed *in vivo*. (B) Overall survival of above was determined by Kaplan-Meier survival curves. A log-rank test was used to assess the statistical significance of differences**.** (C, D) The effect of ACOT12 knockdown on cell metastasis and the effect of Slug on ACOT12 knockdown cell metastasis were assessed *in vivo*. (E) WB detected expression of ACOT12, H3ac, Slug in human ICC tissue and ICC metastasis tissue. SH: shRNA, OE: Overexpression, Data are shown as mean ± SD, significance was determined using the Student's t test or one-way ANOVA. (*P< 0.05, **P< 0.01).

Finally, we validated above findings in ICC xenograft model. We established experimental metastasis model by injecting ICC cells via intraperitoneal cavity. The results showed that compared with HUCCT1 cell control group, an observable decrease in abdominal cavity metastasis of ACOT12 overexpression cell group but no change of ACOT12 overexpression and Slug overexpression HUCCT1 cell group (Fig. 6A). Prognostic analysis indicated that compared with HUCCT1 cell control group an observable increase in survival time of ACOT12 overexpression cell group but no change of ACOT12 overexpression and Slug overexpression cell group (Fig. 6B). We further established another experimental metastasis model by injecting RBE cells directly into liver. The results showed that, compared with control group, an obvious increase of the number of liver metastatic lesions was observed in ACOT12 knockdown group, and that the knockdown of Slug expression dramatically reduced the increased metastasis of ACOT12 knockdown RBE xenograft tumors back to the levels of control group (Fig. 6C, D). WB detected that compared with human ICC tissue, ACOT12 was down-regulated, but H3ac, Slug were up-regulated in human ICC metastasis tissue (Fig. 6E). Taken together, these findings demonstrate that ACOT12 suppresses ICC metastasis *in vivo*.

## Discussion

ICC is the second most common primary hepatic tumor, and accounts for nearly 3% of all gastrointestinal cancers diagnosed worldwide^29^, ^30^. Although incidence rates for ICC vary widely, the highest rates have been reported in Thailand, China, and other parts of Asia^31^. At present, surgical resection results in a better outcome in patients with ICC, however, it still has an extremely poor 5-year survival and high recurrence rate after resection^32^. To solve this problem, it is necessary to explore novel diagnostic biomarkers and to find new therapeutic targets for ICC. Acetyl-CoA metabolic aberration is a direction worth exploring.

Acetyl-CoA is closely related to various cellular progression in cell growth and mitosis. Several researches have shown that acetyl-CoA induced cell growth by promoting the acetylation of histones at genes involved in cell growth and the cell cycle^33^, ^34^. ACOT12 appears to be the major enzyme responsible for hydrolysis of acetyl-CoA^35^, which induces tumor cell metastasis^12^. In present study, we found that ACOT12 inhibited ICC cell metastasis by down-regulating acetyl-CoA level and histone acetylation. We also evaluated five other genes responsible for regulation of acetyl-CoA in paired tissues of ICC and normal tissues from TCGA database, then chose ACLY and ACSS1, which changed most significantly, to validate the association of their expression with ICC, as well as their prognostic values in human ICCs. We found that the expression level of ACLY was higher in ICC tissues than para-tumor tissues (Fig.S1F, H), while the expression level of ACSS1 had no differences between ICC tissues and para-tumor tissues (Fig.S1J, L). And both of them had no significant association with OS (Fig.S1I, M). Furthermore, the remaining genes, although the changes were not the most obvious in ICC, have been reported to participate in other cancer progression^36^, ^37^. They may also play important roles in ICC progression and we will explore in the future.

Slug belongs to the Snail superfamily of transcriptional repressors, which is responsible for regulating the transcriptional repression activity^38^. Recent evidences suggest that Slug participates in epithelial-mesenchymal transition (EMT) during the promotion of cell migration, apoptosis, differentiation by regulating the expressions of its downstream target genes like E-cadherin, N-cadherin, Vimentin etc.^39^-^41^, and therefore can promote cancer invasion and metastasis^42^-^44^. Further study indicated that ACOT12 hindered ICC cell metastasis via inhibition of EMT gene Slug expression. Besides, ACC1 promoted breast cancer metastasis via increase of EMT gene Smad2 expression and ACOT12 hindered HCC cell metastasis via inhibition of EMT gene Twist2 expression^12^, ^17^. From above results, acetyl-CoA metabolic aberration influences cancer metastasis by regulating different EMT genes, and reasons for different EMT genes should be further investigated. Furthermore, the mechanism of ACOT12 down-regulation in ICC is still unclear and need to be explored.

In conclusion, our study revealed ACOT12 as a novel inhibitor of ICC malignant progression. Furthermore, knocking down ACOT12 up-regulated acetyl-CoA level leading to increased H3 acetylation and Slug expression, which promoted ICC metastasis. These data suggest that ACOT12 plays critical roles in tumor metastasis and may become a promising prognostic indicator and potential therapeutic target for ICC metastasis.

## Supplementary Material

Supplementary figures.

## Abbreviations

ICC: intrahepatic cholangiocarcinoma
ACLY: ATP-citrate lyase
ACSS1: acetyl-CoA synthetase 1
ACSS2: acetyl-CoA synthetase 2
PDC: pyruvate dehydrogenase complex
ACOT12: acyl-CoA thioesterase 12
ACC1: acyl-CoA carboxylase1
EMT: epithelial-to-mesenchymal transition
CCK-8: Cell Counting Kit-8
IHC: immunohistochemistry
WB: western blot
qRT-PCR: quantitative real time polymerase chain reaction
TMA: tissue microarray
