# Supplementary material for: ACOT12-mediated acetyl-CoA hydrolysis suppresses intrahepatic cholangiocarcinoma metastasis by inhibiting epithelial-mesenchymal transition
Source: J Cancer. 2022 Mar 14;13(6):1734–44. doi: 10.7150/jca.62169 (PMC8990409; doi:10.7150/jca.62169)
Supplement: Supplementary file 1 — Supplementary figures. [file jcav13p1734s1.pdf]

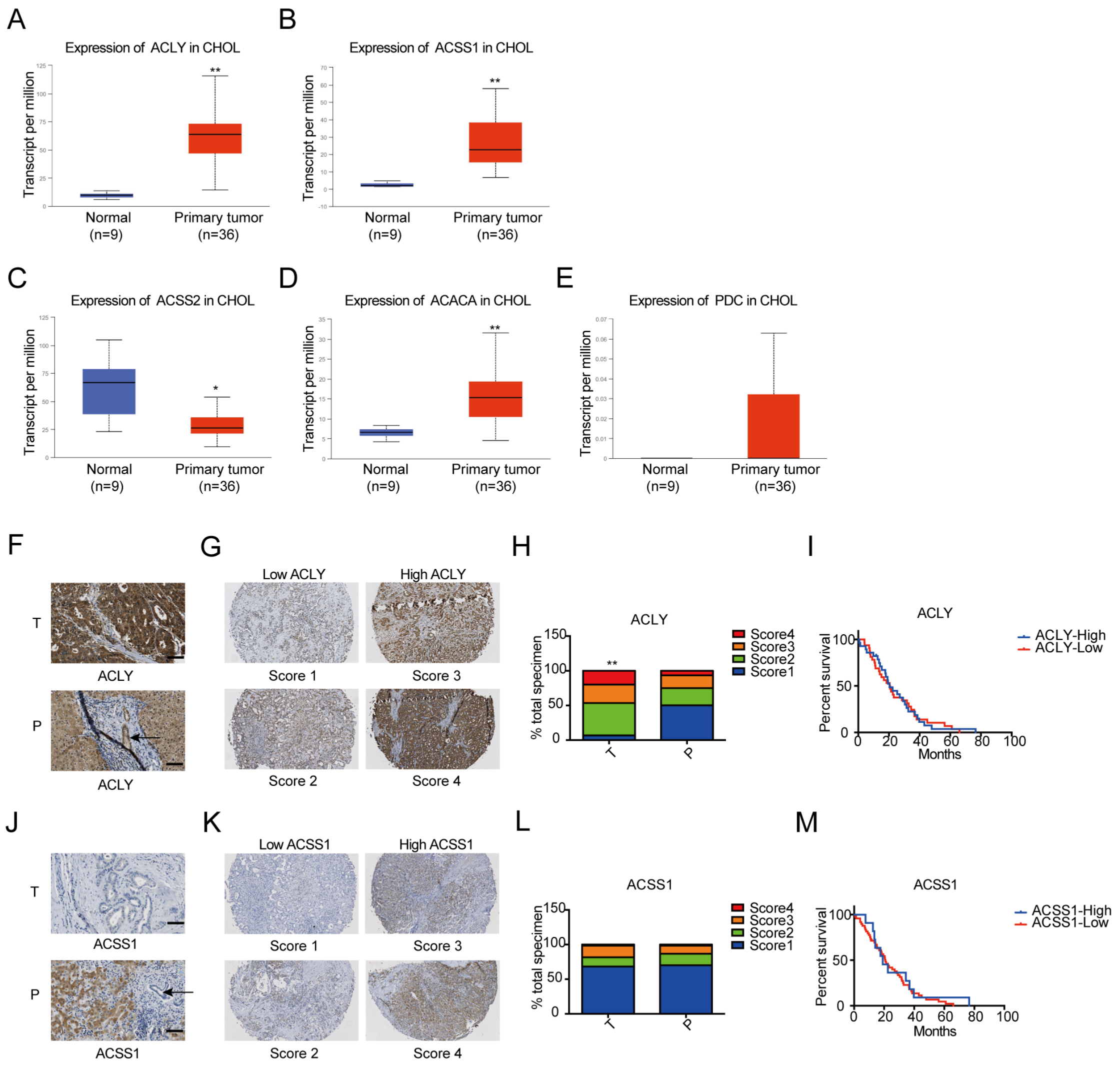

Fig S1 Expression of other genes involved in acetyl-coA metabolism in ICC.

(A-E) Expression of other genes involved in acetyl-coA metabolism in 36 ICC tissues and 9 normal tissues from TCGA database. Significance was determined using the Student's t test.

(F) Representative images of immunohistochemical (IHC) staining of ACLY from one of 60 paired samples of ICC tissues (T) and para-tumor tissues (P) from a tissue microarray. Scale bar, 50μm.

(G) Scores indicate ACLY protein levels in representative tumor tissues.

(H) Quantification of ACLY protein levels according to IHC scores in 60 paired samples of T and P, respectively. Significance was determined using the $\chi 2$ test. Data are shown as percentage of total specimen.

(I) The prognostic significance of ACLY for ICC patients from a tissue microarray analyzed by Kaplan-Meier survival curves. A log-rank test was used to assess the statistical significance of differences.

(J) Representative images of immunohistochemical (IHC) staining of ACSS1 from one of 60 paired samples of ICC tissues (T) and para-tumor tissues (P) from a tissue microarray. Scale bar, 50μm.

(K) Scores indicate ACSS1 protein levels in representative tumor tissues.

(L) Quantification of ACSS1 protein levels according to IHC scores in 60 paired samples of T and P, respectively. Significance was determined using the $\chi 2$ test. Data are shown as percentage of total specimen.

(M) The prognostic significance of ACSS1 for ICC patients from a tissue microarray analyzed by Kaplan-Meier survival curves. A log-rank test was used to assess the statistical significance of differences. (*P< 0.05, **P< 0.01).

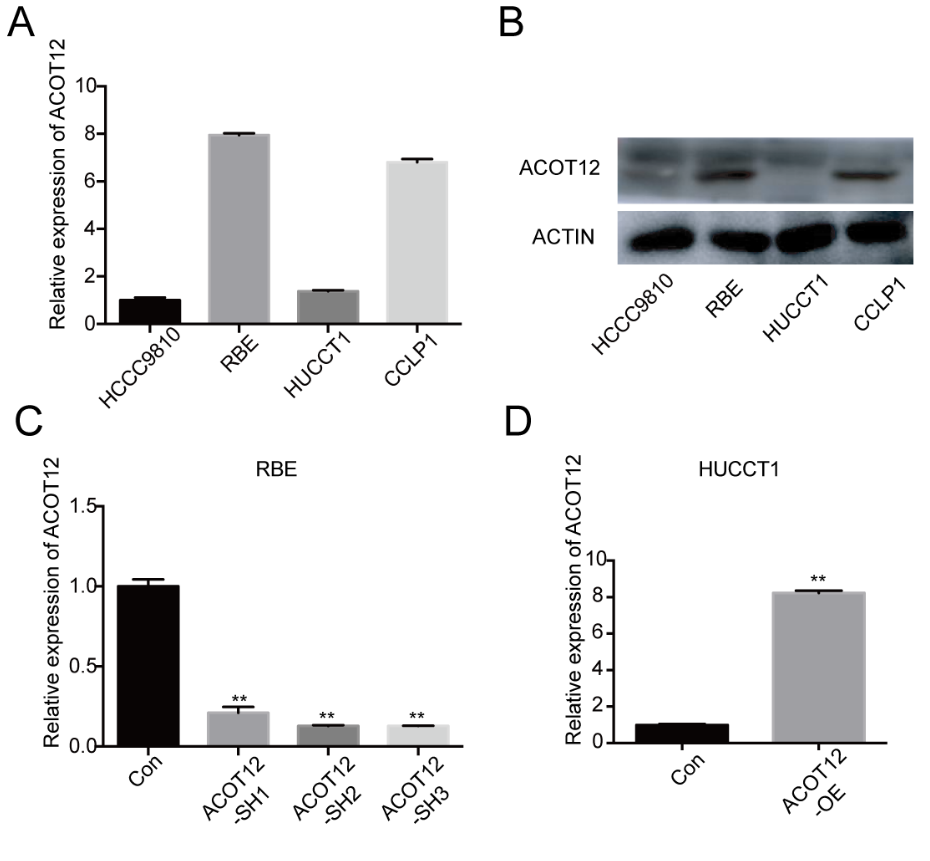

Fig S2 ACOT12 knockdown and overexpression ICC cell lines are constructed.

(A, B) Expression of ACOT12 was detected by qRT-PCR and WB in 4 ICC cell lines.

(C, D) Knockdown or overexpression of ACOT12 in ICC cells was detected by qRT-PCR. SH: shRNA, OE: Overexpression, Data are shown as mean ± SD, significance was determined using the Student's t test. (*P< 0.05, **P< 0.01).

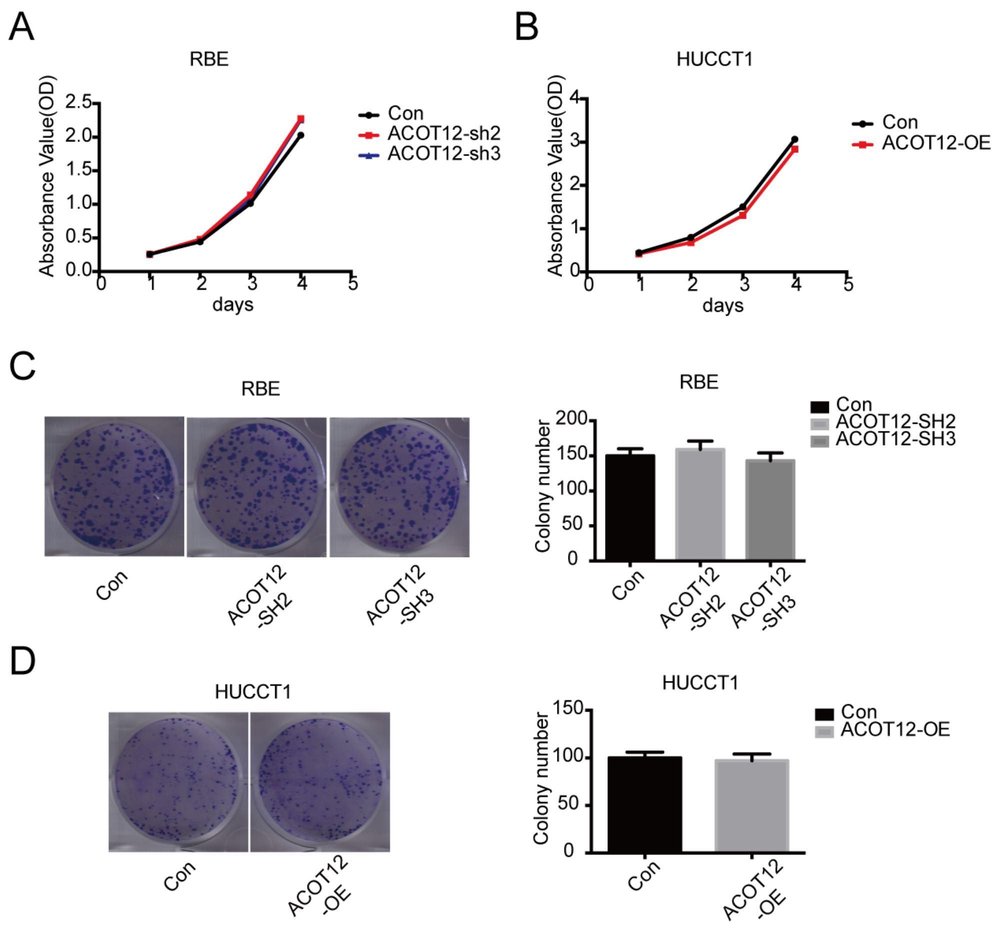

Fig S3 ACOT12 does not affect ICC cells proliferation in vitro.

(A, B) CCK8 assays were employed to detect the proliferation of ICC cells.

(C, D) Colony formation assays were employed to detect the proliferation of ICC cells.

SH: shRNA, OE: Overexpression, Data are shown as mean ± SD.
